# Baroreflex function, haemodynamic responses to an orthostatic challenge, and falls in haemodialysis patients

**DOI:** 10.1371/journal.pone.0208127

**Published:** 2018-12-06

**Authors:** Tobia Zanotto, Thomas H. Mercer, Marietta L. van der Linden, Jamie P. Traynor, Colin J. Petrie, Arthur Doyle, Karen Chalmers, Nicola Allan, Jonathan Price, Hadi Oun, Ilona Shilliday, Pelagia Koufaki

**Affiliations:** 1 Queen Margaret University, Centre of Health, Activity and Rehabilitation Research, Edinburgh, United Kingdom; 2 Renal and Transplant Unit, Queen Elizabeth University Hospital, Glasgow, United Kingdom; 3 Department of Cardiology, Monklands Hospital, Airdrie, United Kingdom; 4 Renal Unit, Victoria Hospital, Kirkcaldy, United Kingdom; 5 Renal Unit, Monklands Hospital, Airdrie, United Kingdom; University Medical Center Utrecht, NETHERLANDS

## Abstract

**Background:**

Stage 5 chronic kidney disease patients on haemodialysis (HD) often present with dizziness and pre-syncopal events as a result of the combined effect of HD therapy and cardiovascular disease. The dysregulation of blood pressure (BP) during orthostasis may be implicated in the aetiology of falls in these patients. Therefore, we explored the relationship between baroreflex function, the haemodynamic responses to a passive orthostatic challenge, and falls in HD patients.

**Methods:**

Seventy-six HD patients were enrolled in this cross-sectional study. Participants were classified as “fallers” and “non-fallers” and completed a passive head up tilting to 60^o^ (HUT-60°) test on an automated tilt table. ECG signals, continuous and oscillometric BP measurements and impedance cardiography were recorded. The following variables were derived from these measurements: heart rate (HR) stroke volume (SV), cardiac output (CO), total peripheral resistance (TPR), number of baroreceptor events, and baroreceptor effectiveness index (BEI).

**Results:**

The forty-four participants who were classified as fallers (57.9%) had a lower number of baroreceptor events (6.5±8.5 vs 14±16.7, p = .027) and BEI (20.8±24.2% vs 33.4±23.3%, p = .025). In addition, fallers experienced a significantly larger drop in systolic (-6.4±10.9 vs -0.4±7.7 mmHg, p = .011) and diastolic (-2.7±7.3 vs 1.8±6 mmHg, p = .027) oscillometric BP from supine to HUT-60° compared with non-fallers. None of the variables taken for the analysis were significantly associated with falls in multivariate logistic regression analysis.

**Conclusions:**

This cross-sectional comparison indicates that, at rest, HD patients with a positive history of falls present with a lower count of baroreceptor sequences and BEI.

Short-term BP regulation warrants further investigation as BP drops during a passive orthostatic challenge may be implicated in the aetiology of falls in HD.

## Introduction

The World Health Organization (WHO) global report on falls prevention in older age [1] states that approximately 30% of people aged 65 years and older experience at least one fall every year, and nearly 50% of all injury-related hospital admissions are attributed to falls. Stage 5 chronic kidney disease (CKD-5) patients undergoing haemodialysis (HD) therapy have also been reported to have a higher risk of falling than the general population [2]. Prospective cohort studies of HD patients, with a 12-month follow-up, report that 26.3% [3] to 47% [4] experience at least one fall per annum. Patients who fell were observed to be at increased risk of adverse outcomes such as admission to nursing homes, higher number and duration of hospitalisations [3] and death [5].

A few prospective cohort studies have explored the association of potential clinical risk factors and falls in CKD-5 patients undergoing HD therapy with physical frailty primarily, older age, comorbidity, previous history of falls, and polypharmacy [2–4, 6] appearing to play a central role in the aetiology of falling. A recent review and summary of published evidence on falls in people with CKD, concluded that very few adequate quality studies in this area exist and many studies present with conflicting findings with regard to the importance of age, gender, different comorbidities, HD therapy and other physical frailty indicators, on the incidence and severity of falls in people with CKD-5 [7].

We already know that aging, history of falls and physical frailty are the most consistent risk factors that stand out from the rest, as predictors of future falls in the general geriatric and CKD population [7]. Moreover, cardiovascular disease (CVD) is the most prevalent comorbidity in the CKD population [8] and indices of poor cardiovascular function such as arterial stiffness [9], impaired blood pressure (BP) responses to a passive orthostatic challenge [10], and antihypertensive drug therapies [11, 12], have been linked to a higher prevalence or incidence of falls in elderly but otherwise healthy individuals. In two prospective cohort studies, a lower pre-dialysis systolic BP was found to be associated with falling status in a group of elderly dialysis patients [4, 13] suggesting that falls might be mediated by low BP spells in these patients. Other researchers suggested that autonomic failure and the significant fluid shifts associated with HD therapy might place HD patients at an increased risk of postural dizziness and hypotensive symptoms, possibly resulting in falls [14]. In addition, Cook et al., [4] reported that 31% of falls experienced by HD patients occurred during the transition from the seated to the upright position, suggesting that abnormal BP regulation, leading to dizziness spells, and potentially orthostatic hypotension (OH), may be implicated in the aetiology of falls in these patients. All these observations lead us to hypothesise that impaired BP regulation particularly during postural changes may be an additional risk factor for falls that further exacerbates the risks coming from physical frailty and chronological aging alone.

The baroreceptor reflex, or baroreflex, is the main physiological mechanism involved in the short-lived haemodynamic responses to change in body position, by regulating BP, heart rate, cardiac output, peripheral resistance, and thus preventing hypotension [15]. This mechanism may be altered in CKD patients, and its impairment has been linked to vascular stiffness, increased cardiovascular risk and all-cause mortality in CKD patients [16, 17]. Despite the association of an impaired baroreflex control with the dysregulation of BP during orthostasis [18], which could lead to hypotensive symptoms and falls, the relationship between baroreflex function and falls in HD patients has been largely unexplored. Therefore, our study is the first step in the process of collecting and documenting evidence of potential relationships between falls and BP control during an orthostatic challenge.

The aims of this study were to explore the hypotheses that impaired baroreflex function would be associated with falls behaviour, amongst HD patients and that self-reported fallers would be more likely to have worse haemodynamic responses to an orthostatic challenge.

## Materials and methods

### Study design

An observational prospective study design was used to explore the relationship between baroreflex function and the falling status (“faller” vs “non-faller”) in a group of prevalent HD patients.

### Setting

The study was conducted in two Renal Units located in North Lanarkshire and Fife, United Kingdom, between October 2015 and August 2018. Recruitment started in October 2015 and continued on a rolling basis until December 2017. All baseline assessments were performed between October 2015 and December 2017, while the follow-up period ran from November 2015 to August 2018.

This research project abided by the ethical principles for medical research involving human subjects, as set out by the world medical association declaration of Helsinki, and received ethical approval by the West of Scotland NHS and Queen Margaret University Research Ethics Committees (NHS REC reference number: 15/WS/0079; ClinicalTrials.gov registration number: NCT02392299).

### Participants

Ambulatory adult (>18 years) haemodialysis patients stable on HD therapy for at least 3 months fluent in spoken and written English were considered eligible to participate in the study.

Exclusion criteria were unstable dialysis and medication treatment, lower limb amputation without prosthesis, unstable cardiac condition, suspected or known aneurysm, clinically severe left ventricular outflow obstruction, critical mitral stenosis, critical proximal coronary artery stenosis, critical cerebrovascular stenosis, pregnancy and severe cognitive impairment.

Eligible patients were provided with a participant information sheet and were given seven days to consider whether to participate in the research project. All patients who agreed to take part provided written informed consent.

### Standardisation of testing procedures

The assessment visit lasted about 2 hours, and occurred on a non-dialysis day, in order to minimise the influence of fluid and electrolyte shifts on data collected. Participants were instructed to follow standardised assessment protocol procedures that included no meals, caffeine or alcohol-containing drinks for at least 2 hours before the assessment, no smoking and no unaccustomed physical exercise on the 24 hours preceding testing. No changes to medication prescription and timings were imposed.

### Sociodemographic characteristics

Participant demographics (age, gender, height, weight, body mass index), and clinical characteristics (dialysis vintage, Charlson comorbidity index, medications and blood biochemistry data) were obtained from the patients’ medical records. Height and weight were measured on the day of assessment.

### Falls

A fall was operationally defined as an unexpected event in which the participant comes to rest on the ground, floor, or lower level [19]. The researcher (TZ) administered a falls questionnaire to all participants during dialysis, once a month, for a period of 12 months. The number of falls, circumstances, location, activities, precipitating factors, injuries, actions were documented for every fall. In addition, a history of falls questionnaire was completed by every participant at the baseline assessment visit. Participants were asked to report any falls they might have had in the previous 12 months. We defined as “faller” everyone who met at least one of these conditions: 1) at least one self-reported fall in the previous 12 months, and/or 2) at least one fall recorded by the researcher during the prospective follow-up period.

### Haemodynamic and baroreflex function

The haemodynamic and baroreflex function was assessed at rest, in the supine position, and in response to a passive orthostatic challenge that involved head up tilting to 60 degrees from the supine position (HUT-60^o^).

For this measurement, the participants lay quietly awake in the supine position for 15 minutes [20], and were then tilted up for 5 minutes by means of an electrically controlled bed, followed by another 5 minutes of supine rest. The Task Force Monitor 3040i (CNSystems, Graz, Austria), was used for the non-invasive measurement of all hemodynamic data [21, 22, 23]. Stroke volume (SV), cardiac output (CO), and total peripheral resistance (TPR) were recorded by means of impedance cardiography (ICG). Heart rate (HR), R-R interval (RRI), and continuous BP (contBP) were measured by means of 6-lead electrocardiography (ECG) and continuous photoelectric plethysmography. The contBP was measured from the index or middle finger, based on which finger returned the best BP reading, by means of the unloading technique [24], and it was calibrated against oscillometric BP measurements. The hydrostatic effects of tilting were corrected by keeping the contBP monitor at heart level throughout the measurement, as per manual instructions. Oscillometric BP (oscBP) was measured with an electronically controlled sphygmomanometer connected to the participants’ arm that was free from arteriovenous fistulas.

Baroreflex function was assessed by means of the baroreceptor effectiveness index (BEI), which represents how often the baroreflex produces a change in HR in response to a perturbation in BP [25]. The Task Force Monitor assesses the spontaneous activity of baroreceptors by using the sequence method which has been described to provide the equivalent prognostic information of the invasive methods used to measure the baroreflex [26].

The following variables were also derived and included in the analyses: i) blood pressure (BP) ramps defined as either an increase (up-ramp) or decrease (down-ramp) in contBP of at least 1 mmHg for 3 consecutive heart beats: “total-ramps” were defined as the sum of all down-ramps and up-ramps, ii) baroreceptor events, defined as the simultaneous coupling of a BP ramp with either an increase or decrease of the RRI of at least 4 ms. More precisely, a “down-event” was classified as a concomitant decrease of continuous systolic BP (contSBP) and RRI of at least 1 mmHg and 4ms respectively, while an “up-event” was classified as a concomitant increase of contSBP and RRI of at least 1 mmHg and 4ms respectively. “Total-events” were classified as the sum of all down-events and up-events iii) The BEI was then computed as the ratio of occurred baroreceptor events and detected BP ramps expressed as a percentage. This index can be characterised by three components: the “down-BEI” that represents the ratio of occurred down-events and detected down-ramps, the “up-BEI” that represents the ratio of occurred up-events and detected up-ramps, and the “total-BEI” that represents the ratio of occurred total-events and detected total-ramps. In addition, the baroreflex sensitivity (BRS) was automatically computed by the Task Force Monitor software as the average slope of the regression lines between the RRIs and the contSBP values resulting from every baroreceptor event [27].

### Statistical analysis

Statistical analyses were performed with SPSS (Version 23.0 for Windows, SPSS Inc., Chicago, IL). The Shapiro-Wilk Test (S-W) was used for the normal distribution checks of all data. Differences between fallers and non-fallers in demographic and clinical characteristics were analysed by means of a Chi-Squared test for categorical variables, and by either Mann-Whitney U or independent t-tests, as appropriate, for continuous variables: results are expressed as mean and standard deviation (SD).

The effect of grouping, i.e. fallers vs non-fallers, on the baroreflex and haemodynamic variables was analysed by means of either parametric (independent t-tests) or non-parametric (Mann-Whitney U) independent comparisons, based on normal distribution assumptions. Statistical limits for interpretation were set at an alpha level of *p* = .05.

The association between the baroreflex function/haemodynamic resposes and falls (yes or no) was analysed by means of logistic regression analysis: variables reaching a statistical significance level of p≤ 0.10, in the preliminary independent comparisons, were entered in a univariate logistic regression model, which was adjusted a posteriori in a multivariate analysis. Statistical limits for interpretation of the logistic regression analysis were also set at an alpha level of p = .05.

## Results

### Recruitment and loss to follow-up

Three hundred and five patients undergoing outpatient HD therapy at the Renal Units were screened for eligibility by members of the renal team. Of these, 215 patients were deemed eligible to participate and therefore approached for recruitment and consenting. The recruitment rate was 35.3%, with 76 patients agreeing to participate in the study, and completing all baroreflex and haemodynamic measurements. Nine patients (11.8%) were lost to follow-up due to renal transplantation (n = 3; 3.9%) and death (n = 6; 7.9%), although 5 of these patients were retained in the data analysis due to their positive history of falls. Moreover, 14 patients were excluded from the baroreflex function data analysis due to atrial fibrillation (n = 7; 9.2%) and to poor circulatory blood flow to the fingers, which rendered the contBP measurement unusable (n = 7; 9.2%). This resulted in the inclusion of 62 patients in the baroreflex function analysis. After the exclusion of the 7 patients with poor blood circulation, 69 patients were retained for the haemodynamic responses analysis.

### Sociodemographic characteristics

The demographic and clinical characteristics of patients are summarised in Table 1. Fallers were more likely to have diabetes as primary renal disease (PRD), and less likely to use diuretics compared to non-fallers.

**Table 1:** Sociodemographic and clinical characteristics of study participants (mean ± standard deviation).

Variables	All patients (76)	Fallers (44)	Non-fallers (32)	P-value
**Sociodemographic characteristics**				
Sex (% M)	53.9	52.3	58.1	0.620
Age (years)	61.1±14	59.9±13.2	62.3±15.2	0.482
Weight (Kg)	79.7±18.3	77.4±18.8	83.1±17.7	0.117
Height (cm)	165.8±8.7	166.4±9.7	165.3±7.2	0.595
BMI (Kg * m^-2^)	29±6.3	28±6.7	30.3±5.6	0.131
**Clinical history**				
Dialysis vintage (days)	726±716	755±777	666±633	0.780
CCI (score)	5.2±2.3	5.2±2.1	5.2±2.6	0.841
Primary renal disease (%)				
*Diabetic nephropathy*	26.7	34.9	12.9	0.033
*Glomerulonephritis*	18.7	18.6	19.4	0.935
*Polycystic kidney*	12	2.3	25.8	0.002
*Renovascular or hypertensive*	8	4.7	12.9	0.199
*Other*	18.7	20.9	16.1	0.603
*Uncertain aetiology*	17.3	18.6	12.9	0.512
Type of vascular access (%)				
*Arteriovenous fistula*	66.2	62.8	71	0.463
*Central-venous*	33.8	37.2	29	0.463
Inter-dialytic weight gain (Kg)	1.5±1.3	1.6±1.4	1.5±1.2	0.849
**Prescribed medications**				
Medications (n°)	11.8±3.7	12.3±3.8	11±3.7	0.106
Beta blockers use (%)	49.3	43.2	56.7	0.255
ACE-inhibitors use (%)	8	4.5	13.3	0.174
Ca-channel blockers use (%)	56	59.1	53.3	0.624
AngII-receptor antagonists use (%)	16	15.9	16.7	0.931
Alpha blockers use (%)	29.3	36.4	20	0.131
Antihypertensive use (%)	84	81.2	86.7	0.579
>1 antihypertensive use (%)	50	50	50	1.000
Opiates use (%)	20	15.9	26.7	0.258
Antidepressants use (%)	32	38.6	23.3	0.167
Diuretics use (%)	37.3	27.3	53.3	0.023
**Laboratory values**				
Hb (g/dL)	11.2±1.2	11.2±1.1	11.2±1.2	0.763
CRP (mg/L)	24.3±43.6	28.7±49.8	17.6±33.4	0.083
Bicarbonate (mmol/L)	21.2±3.2	21.4±3.3	20.8±3	0.455
Na (mmol/L)	139±2.8	138.7±3	139.4±2.5	0.728
K (mmol/L)	4.6±0.7	4.7±0.7	4.5±0.6	0.690
Urea (mg/dL)	16.3±5.1	16.2±5.9	16.4±4.1	0.859
Phosphate (mmol/L)	1.5±0.6	1.5±0.6	1.5±0.5	0.894
PTH (ρmol/L)	27.5±31.3	27.3±34.2	27.9±27.9	0.859
Albumin (g/L)	37.1±4.2	36.8±4.5	37.5±3.8	0.435
Adjusted calcium (mmol/L)	2.3±0.1	2.3±.01	2.4±0.1	0.983
URR (%)	71.2±6	71.9±6.5	70.1±5.1	0.205
Kt/V	1.4±0.3	1.4±0.3	1.3±0.2	0.167
Creatinine (μmol/L)	634.3±159.9	617.4±173.7	654.6±139.6	0.326

**Abbreviations**: BMI: body mass index; CCI: Charlson comorbidity index; ACE: angiotensin-converting enzyme; Ca: calcium; AngII: angiotensin II; Hb: hemoglobin; CRP: C-reactive protein; Na: sodium; K: potassium; PTH: parathyroid hormone; URR: urea reduction ratio.

### Falls

During the 12-month follow-up, 26 of 72 patients (36.1%) experienced at least one fall, of which 14 (53.8%) experienced multiple falls. The maximum amount of falls experienced by one patient was 21. A total of 80 falls were recorded, resulting in an incidence of 1.11 falls/patient-year. In addition, 33 of 76 patients (43.4%) reported falling at least once in the previous 12 months and, overall, 44 of 76 patients (57.9%) reported either a fall in the previous year or during follow-up, and were therefore classified as fallers.

The most commonly reported factors perceived as a contributing cause of the falls experienced during follow-up were gait and balance issues (65.4%), environmental hazards (46.2%), and dizziness or syncope-like events (42.3%).

### Haemodynamic and baroreflex function

The differences between fallers and non-fallers in all baroreflex variables are summarised in Table 2. At rest, fallers had a statistically significant lower count of baroreceptor “down-events” and “total-events”, which also resulted in a significant lower “down-BEI” and “total-BEI”, compared to non-fallers. In addition, the “up-BEI” during HUT-60° was also significantly lower in fallers. No significant differences in BRS were detected between the two groups.

**Table 2:** Baroreflex function: Differences between fallers and non-fallers (mean ± standard deviation). Group means reflect averaged data for the total duration of 5 minutes in each postural position.

	Supine		HUT-60	
Variables	Fallers	Non-fallers	Fallers	Non-fallers
Up-ramps (n°)	20.6±21	19.5±13.5	23.2±15.9	17.8±13.5
Down-ramps (n°)	18.9±17.7	17.4±11.9	22.3±14.2	16.6±12.9
Total-ramps (n°)	39.5±38.2	36.8±25	45.5±29.2	34.5±26
Up-events (n°)	3.1±4	6.8±9.5	2.3±3.5	3.6±4.4
Down-events (n°)	3.4±4.8*	7.1±7.8	3±3.6	3.9±4.5
Total-events (n°)	6.5±8.5*	14±16.7	5.2±6.3	7.5±8.4
Up-BEI (%)	15.5±20.1	29.2±29.4	10.7±13.6*	19±15.7
Down-BEI (%)	23.3±27*	36.6±22.8	13.5±15.7	19.2±15.7
Total-BEI (%)	20.8±24.2*	33.4±23.3	12.6±13.4	19.1±13.2
BRS (ms/mmHg)	9.2±8.3	10±6.1	6.8±4.9	9.8±8.3

**Abbreviations**: HUT-60: head-up tilt at 60°; Up-BEI: up-events baroreceptor effectiveness index; Down-BEI: down-events baroreceptor effectiveness index; Total-BEI: total-events baroreceptor effectiveness index; BRS: baroreflex sensitivity

* indicates a statistical significant difference between groups (p < .05).

The haemodynamic variables of fallers and non-fallers, in the supine position and during HUT-60°, are described in Table 3. The differences in SV, CO, TPR, HR, contSBP, contDBP, OscSBP, and OscDBP from the supine position to HUT-60° are expressed as absolute values. A significant larger decrement of OscSBP and OscDBP from supine to HUT-60° was detected between fallers and non-fallers, while no differences in the remaining haemodynamic variables were found.

**Table 3:** Haemodynamic variables: Differences between fallers and non-fallers (mean ± standard deviation).

	Supine		HUT60		ΔSupine-HUT60	
	Fallers	Non-fallers	Fallers	Non-fallers	Fallers	Non-fallers
RRI (ms)	869.2±134.1	926.6±187.5	809.4±168	868.4±192.8	-57.9±70.2	-58.1±64.5
HR (bpm)	70.9±10.7	67.7±13.2	77.7±15.2	72.9±15.2	6.6±8.3	5.5±5.8
contSBP (mmHg)	125.4±23.5	122±21.6	126.6±21.8	125.1±20.3	3.5±15.6	3±8
contDBP (mmHg)	76.5±14	79.4±16.4	82±15.2	85.4±16.3	6.1±10.2	6±7.6
contmBP (mmHg)	97.3±10.3	97±18.3	100.8±17.7	101.9±17.9	4.7±12.1	4.9±7.3
SV (ml)	63.6±14	69.1±16.1	59.1±11.8	62.9±15.5	-4.1±12.9	-6.2±16.3
CO (L/min)	4.5±1.1	4.7±1.5	4.5±0.9	4.5±1.2	0.03±0.9	-0.2±1.2
TPR (dyne*s/cm^5^)	1731.8±432.4	1763.9±610.8	1797.7±451.5	1919.5±583.1	77.9±347.2	155.5±416.4
SI (ml/m^2^)	34.8±8.3	37±10.7	32.4±7.4	33.4±8.5	-2.2±7	-3.7±9.1
CI (L/min*m^2^)	2.5±0.7	2.5±1	2.5±0.5	2.4±0.7	0.01±0.5	-0.1±0.6
TPRI(dyne*s*m^2^/cm^5^)	3168.5±789.6	3381.1±1340.9	3292.1±806.8	3645.1±1259.6	146.7±624	264.1±773.5
TFC (1/kOhm)	32.3±10.6	34.4±11.1	30.3±10.1	32.4±11.1	-1.6±1.7	-2±1.8
OscSBP (mmHg)	131.3±22.6	124.1±19.8	122.2±18.3	123.9±13.1	-6.4±10.9*	-0.4±7.7
OscDBP (mmHg)	81.9±12.9	79.9±15.8	79.1±13.2	81.6±17.4	-2.7±7.3*	1.8±6

**Abbreviations**: HUT60: head-up tilt at 60°; RRI: R-R interval; HR: heart rate; contSBP: continuous systolic blood pressure; contDBP: continuous diastolic blood pressure; contmBP: continuous mean blood pressure; SV: stroke volume; CO: cardiac output; TPR: total peripheral resistance; SI: stroke index; CI: cardiax index; TPRI: total peripheral resistance index; TFC: thoracic fluid content; OscSBP: oscillometric systolic blood pressure; OscDBP: oscillometric diastolic blood pressure; ΔSupine-HUT60 represents the difference between the variables averaged over 5 minutes of HUT-60° and the variables averaged over 5 minutes of supine recording

* indicates a statistical significant difference between groups (p < .05).

### Factors associated with falls

In univariate logistic regression, diabetic nephropathy, number of “down-events” and “total-events” in the supine position, “up-BEI” in the supine position, “up-BEI” in HUT-60°, OscSBP and OscDBP difference from the supine position to HUT-60° were associated with increased odds of falling (Table 4).

**Table 4:** Logistic regression analysis: Factors associated with falls.

	Univariate		Adjusted	
Factors	Odds Ratio (95% CI)	P-value	Odds Ratio (95% CI)	P-value
**Clinical characteristics**				
Diabetic nephropathy (%)	3.616 (1.064–12.286)	0.039	-	-
**Baroreflex function**				
Down-events supine (n°)	0.909 (0.832–0.993)	0.034	0.932 (0.851–1.021)	0.130
Total-events supine (n°)	0.953 (0.910–0.997)	0.037	0.961 (0.919–1.006)	0.087
Up-BEI supine (%)	0.977 (0.956–0.999)	0.045	0.978 (0.954–1.001)	0.066
Up-BEI HUT-60 (%)	0.961 (0.925–1.000)	0.048	0.975 (0.936–1.015)	0.221
Down-BEI supine (%)	0.980 (0.959–1.001)	0.058	0.986 (0.964–1.008)	0.216
Total-BEI supine (%)	0.978 (0.956–1.001)	0.060	0.983 (0.960–1.008)	0.175
Total-BEI HUT-60 (%)	0.964 (0.925–1.005)	0.085	0.983 (0.940–1.027)	0.437
**Haemodynamic variables**				
OscSBP Δsupine–HUT60 (mmHg)	0.930 (0.871–0.992)	0.028	0.939 (0.876–1.008)	0.080
OscDBP Δsupine–HUT60 (mmHg)	0.894 (0.813–0.983)	0.021	0.908 (0.816–1.010)	0.075

**Abbreviations**: CI: confidence interval; Up-BEI: up-events baroreceptor effectiveness index; Down-BEI: down-events baroreceptor effectiveness index; Total-BEI: total-events baroreceptor effectiveness index; HUT60: head-up tilt at 60°; OscSBP: oscillometric systolic blood pressure; OscDBP: oscillometric diastolic blood pressure; ΔSupine-HUT60 represents the difference between the variables averaged over 5 minutes of HUT-60° and the variables averaged over 5 minutes of supine recording.

The univariate analysis was adjusted for diabetic status, as we retrospectively identified this factor to be potentially a significant confounder of the study results (Table 1). In this multivariate logistic regression model, none of the variables were significantly associated with falling (Table 4).

### Further analyses

**Fig 1:**
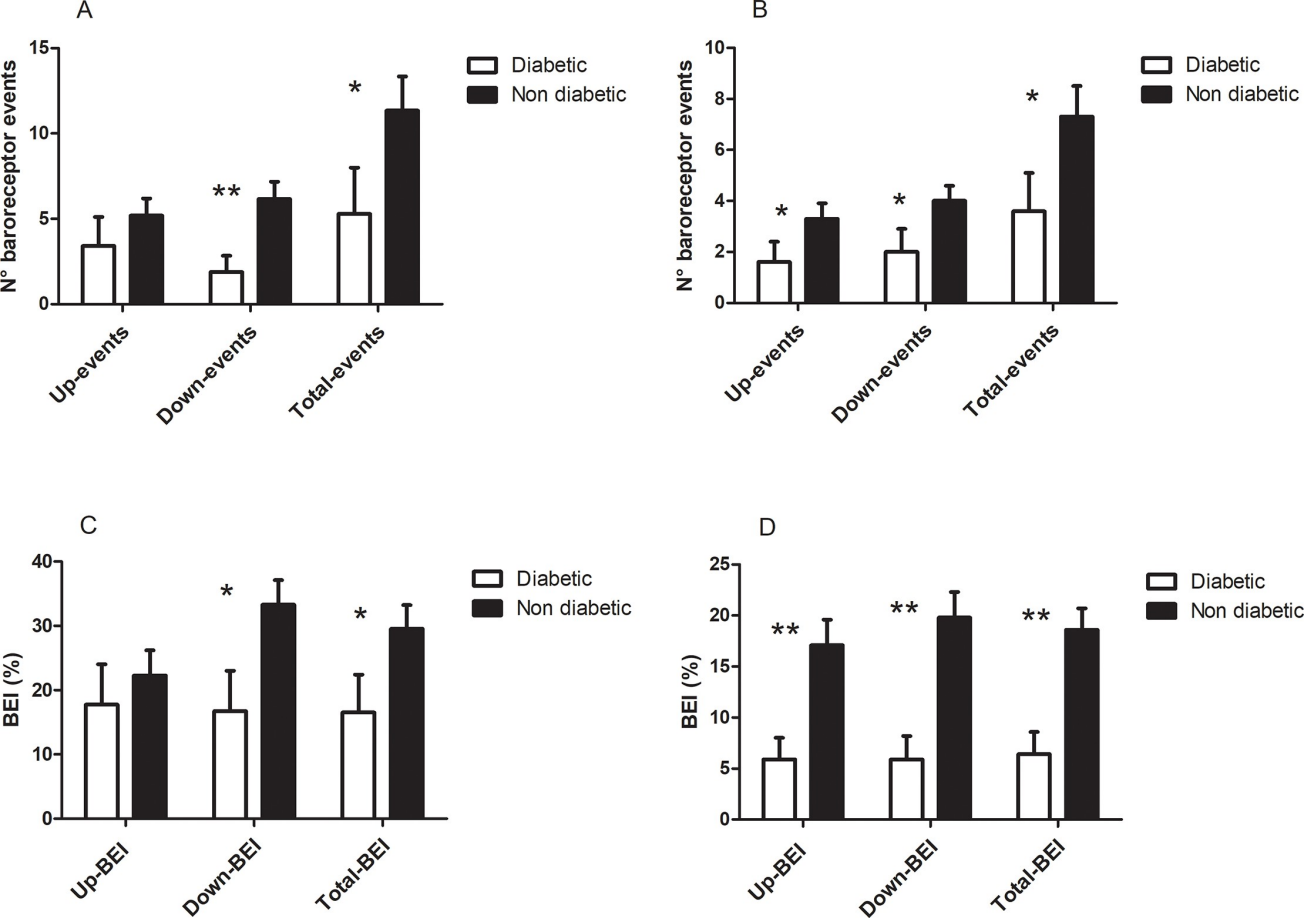
Baroreflex function in diabetic vs non-diabetic patients. Fig 1A shows the number of baroreceptor events in the supine position; Fig 1B shows the number of baroreceptor events in HUT-60°; Fig 1C shows the baroreceptor effectiveness index (BEI) in the supine position; Fig 1D shows the BEI in HUT-60°. * indicates a statistically significant difference (p< .05). ** indicates a statistically significant difference (p< .01).

**Fig 2:**
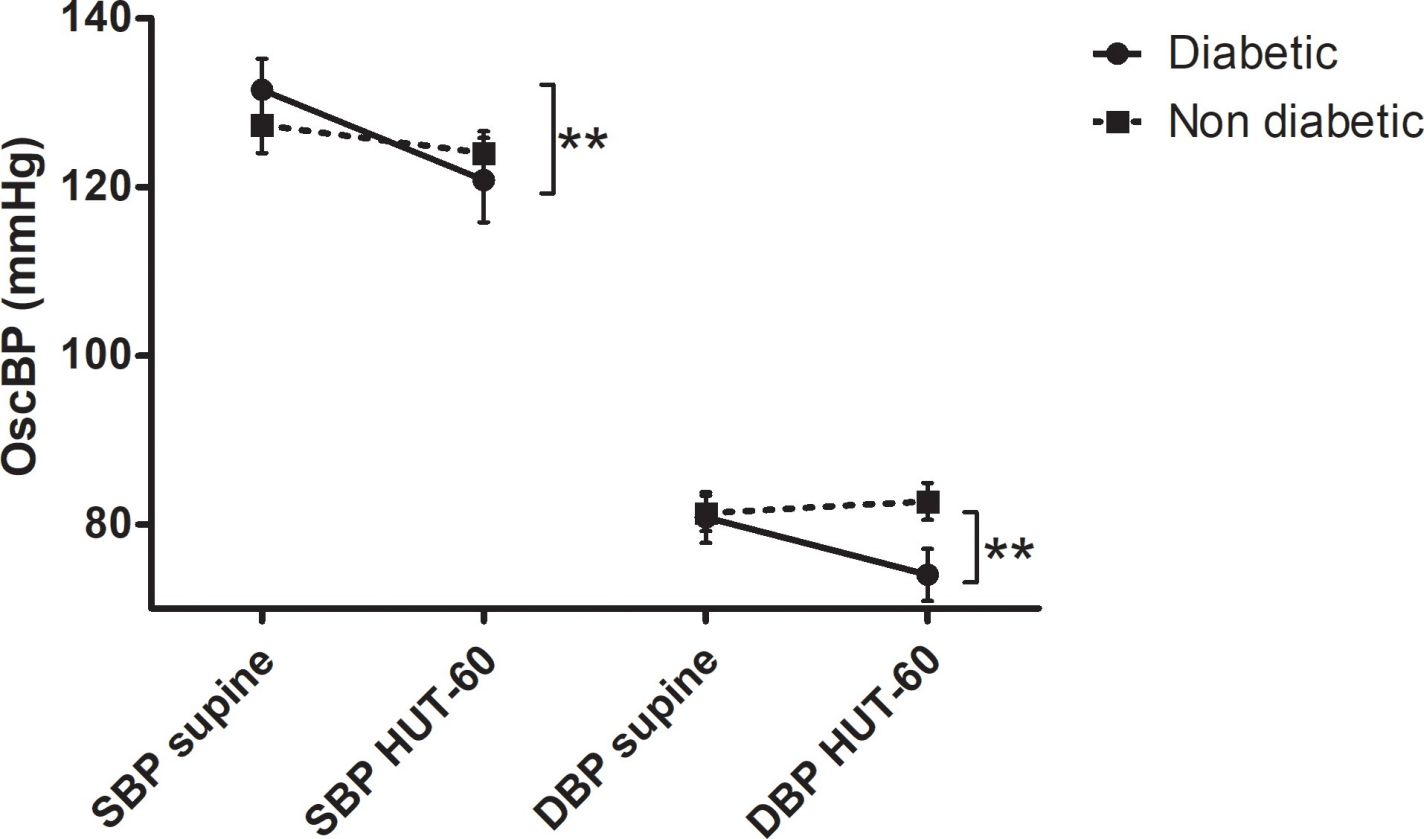
Changes in systolic (SBP) and diastolic (DBP) oscillometric blood pressure (OscBP) during transition from the supine position to HUT-60° (diabetic vs non diabetic). ** indicates the statistically significant drop in OscBP in diabetic patients (p< .01).

In order to evaluate the weight of the confounding effect of diabetes on the study results, we compared diabetic vs non-diabetic patients in terms of baroreflex function and BP response to HUT-60°. The independent comparisons between the two groups indicate that these variables were markedly decreased in diabetic patients (Figs 1 and 2).

In addition, we also performed a point biserial correlation analysis in the sub-group of non-diabetic patients (N = 44) to explore the relationship between the factors entered in logistic regression analysis and falls. No significant correlations were found for any of the baroreflex function/haemodynamic variables and falls (-0.223 ≤R_s_ ≤ -0.088; 0.151 ≤P-values≤ 0.583) when diabetic patients were removed.

The heart rate variability (HRV) characteristics of the study participants are also summarised in S1 Table.

## Discussion

We hypothesised that HD patients classified as fallers would have worse baroreflex function than patients free from falls. In addition, we hypothesised that patients with falls would have worse haemodynamic responses to an orthostatic challenge.

We found that at rest, fallers had lower counts of baroreceptor “down-events” and “total-events”, as well as a lower down-BEI and total-BEI compared to non-fallers. Although we also expected to see a significantly impaired ability to effectively regulate the haemodynamic variables via the arterial baroreflex mechanism in the fallers group, in response to a passive orthostatic challenge, this was not confirmed. However, we noted a significantly larger drop in OscBP during the transition from supine to HUT-60^o^ which warrants further investigation.

Our findings on baroreflex function suggest that a lower number of baroreceptor sequences might discriminate patients with falls from those who are falls-free. Although no differences in the baroreflex slope, as assessed by BRS, were detected between fallers and non-fallers, measures reflecting how often the baroreflex is activated, such as the number of “down-events” and the “total-events”, among other BEI indices, were significantly lower in the group of fallers. Interestingly, in resting conditions, the baroreceptor down-regulation seemed to better discriminate fallers from non-fallers. A baroreceptor down-event occurs when a systolic BP drop is coupled with a concomitant decrease of the RRI, namely an increase in HR. This is a physiologic response to a spontaneous perturbation of BP, which allows the maintenance of haemodynamic homeostasis [15]. Therefore, the lower count of baroreceptor “down-events” observed in fallers, as well as the lower “down-BEI” might indicate a relationship between the failure to increase HR in response to a spontaneous drop in BP and falls.

It should also be noted that, even though we did not assess a control group of healthy participants, the BEI indices measured in our patients (20.8±24.2% in fallers, and 33.4±23.3% in non-fallers) are considerably lower than the average 58±20% BEI measured in healthy individuals [27], while their BRS values were only slightly inferior (-15% to -25%) to those of an age-matched healthy population [28]. Because a reduced BEI has already been shown to be an independent predictor of all-cause mortality in patients with CKD [17], it is possible that this index might predict other adverse outcomes such as falls in this population. Potentially, the lower BEI as well as the lower number of baroreceptor events could be linked to syncope-related falls due to an impaired homeostasis of the HR and BP responses, which may lead to cerebral hypoperfusion with sudden onset of dizziness and pre-syncopal symptoms, which are commonplace among HD patients [14]. Interestingly, in the current study, almost half of the patients who experienced falls during the prospective observational follow-ups (42.3%) reported dizziness or syncope-like events as one of the symptoms preceding a falling event, which indirectly implicates this mechanism in the aetiology of falls in HD patients.

Although a direct biologic mechanism may exist between baroreflex function and falls, given the relationship between impaired baroreflex function and orthostatic BP decrements [18], the study results do not seem to fully support the hypothesis that poor baroreflex function and orthostatic BP regulation are independent risk factors for falls in HD patients. While several baroreflex indices, as well as OscBP, were associated with falls in univariate logistic regression, adjusting the model for diabetic status resulted in no significant association between the baroreflex function/haemodynamic responses and falls.

The role of diabetes, in the context of our study, plays a crucial role as 34.9% of the patients classified as fallers had diabetic nephropathy as PRD, compared to only 12.9% in the group of non-fallers. Diabetic nephropathy represents an advanced stage of diabetes, which is commonly associated with cardiovascular autonomic neuropathy and chronic sympathetic over-activity, both of which can affect the baroreflex and potentially the haemodynamic responses to orthostasis [29]. Therefore, the higher proportion of diabetic patients amongst fallers is likely to be a main driver of the significant differences observed between fallers and non-fallers in terms of baroreflex function and BP response to orthostasis.

The point biserial correlation analysis performed in the subgroup of non-diabetic patients did not reveal any significant correlations between any of the baroreflex/haemodynamic variables and falls, which highlights the mediating effect of diabetes on the study results.

This is an interesting finding considering that diabetes has been found to be an independent risk factor for falls in HD patients [2], and our study results seem to indirectly suggest that impaired baroreflex and BP dysregulation may be one of the biological mechanisms underlying the higher occurrence of falls amongst diabetic HD patients.

Surprisingly, we did not find any differences in the SV, CO, TPR, HR, contSBP, and contDBP responses to the HUT-60° between fallers and non-fallers. This lack of effect may be explained in light of the relatively short duration of the orthostatic challenge. Although 5 minutes of orthostasis are considered to be sufficient for the diagnosis of orthostatic hypotension, according to the current guidelines [20], it is possible that a longer orthostatic challenge could have yielded different results. For instance, Shaw et al., [10] examined the cardiovascular responses to orthostasis in a group of elderly residents in long-term facilities. They found that, during an orthostatic challenge, the decreases in contBP were larger in those with a history of falls, but only in the delayed phase of orthostasis (3–15 minutes) rather than at the initial phase (0–3 minutes). This might explain why we found a significant larger decrement in OscBP, but not in contBP between fallers and non-fallers: whilst OscBP assessment consists of single measurements, which capture the BP at a single time-frame, contBP may provide more useful information than single sphygmomanometer assessments, in terms of actual beat-to-beat variations of BP [30], but its measurement represents an average of several measurements over a given interval of interest. Therefore, the two type of BP measurements, despite being performed in the same phases, do not represent exactly the same haemodynamic data.

During HUT-60°, for instance, the contSBP and contDBP reflect the overall BP performance over the 5 minutes of data acquisition and it is possible that a longer recording interval may also have revealed a larger decrement of BP in fallers. Moreover, the discrepancy between contBP and OscBP measurements during HUT-60° could also be explained in light of a possible hydrostatic effect: because postural changes can modify the distribution of hydrostatic pressures in fluid-filled body compartments [31], it is possible that the transition from supine to HUT-60° may have influenced to some extent the response of contBP due to the initial gravitational shift. On the other hand, during HUT-60°, OscBP was measured when the patient was already in the upright position, and therefore this measurement would be less subjected to hydrostatic adjustments arising from the tilting procedure. Although we sought to minimise the hydrostatic effects of tilting by standardising the testing procedures, as described in S1 Protocol, it is possible that these may have played a role in the discrepancy observed between the two kinds of BP assessments.

It should also be acknowledged that the resting BP of the study participants was surprisingly low considering that HD patients are usually hypertensive. This relatively low BP may be explained in light of the strict testing standardisation procedures which were designed to ensure the best possible haemodynamic state balance (e.g. no caffeine, supine rest prior to the assessment, non-dialysis day), and also by a possible underestimation of BP from the Task Force Monitor [32]. Although this should not affect the study results, since the research aim was focused on exploring the relationship between the relative change in BP and falls, rather than the absolute values of BP, the generalisability of the study results to patients with higher or more poorly controlled resting BP should be cautious.

Only a few studies examined the BP changes in response to orthostasis in HD patients, and found no association between the BP response to a pre-dialysis [4] or post-dialysis [2, 33] orthostatic assessment and the patients’ falling status. Nevertheless, these studies assessed the BP response by means of OscBP measurements after active standing, a procedure that may be subjected to standardisation issues compared to the head-up tilt test, which is considered the reference standard for the assessment of orthostatic hypotension [34].

In addition, it should be acknowledged that the tilting angle might also be partly responsible for the lack of response. Typically, angles of 60°-90° are widely implemented in clinical practice [35] and thus tilting patients beyond 60° could have constituted a larger haemodynamic challenge and concomitant response.

The incidence of falls recorded was 1.11 falls/patient-year and is approximately 2.3 times greater than seen in the non-uraemic, community-dwelling elderly [36]. This confirms the increased risk of falling of HD patients compared with the general healthy population [2].

Although the current study was conducted in a small cohort of patients, our findings relating to the incidence of falls are broadly in agreement with those of larger observational studies. In particular, Desmet et al., [2], reported a yearly incidence of 1.18 falls/patient-year for their HD patients, which is very similar to that observed in our study (1.11 falls/patient-year). Additionally, the proportion of patients observed in our study, who experienced at least one fall during the 12-month follow-up (36.1%), is also very similar to that reported in previous research (28.3%) [6]. Therefore, our findings on falling behaviour in HD patients seem to be representative of this patient group, and results from this study may be generalised to the general population of CKD-5 patients undergoing HD therapy.

### Limitations

First of all, the classification of patients in fallers and non-fallers was based on self-reported information. As previous research has highlighted how recalling information about falls might be subjected to misreporting [37], this could have resulted in some degree of misclassification in the group allocation. We sought to minimise this bias by following up prospectively the participants every month [38], although patients were also classified as fallers if they had experienced at least one fall in the previous 12 months: this kind of information is theoretically more susceptible to misreporting given the longer recall interval [39]. The decision to classify the patients with a previous history of falls also as fallers, regardless of the occurrence of any new fall event during the observational follow-up, was made to counterbalance another risk of bias, namely that of blindly assuming that all patients were free from the clinical outcome of interest, i.e. falls, at the beginning of the study.

In addition, the relatively small sample size did not allow the application of a more exhaustive, a priori, multivariate logistic regression analysis to more robustly test the interrelationships between baroreflex function, haemodynamic responses, and falls.

### Conclusions

This study indicates that, at rest, HD patients classed as “fallers” present with worse baroreflex indexes reflecting how often the baroreflex is activated, as highlighted by the lower number of baroreceptor-mediated sequences of coupled HR and BP. Additionally, a significantly larger decrement of OscBP was observed in “fallers”, even though other haemodynamic responses to HUT-60° were not seen to differ between fallers and non-fallers. Patients with falls were also more likely to have diabetes as PRD, and the diabetic status seems to at least partly mediate the relationship between baroreflex function/BP responses to orthostasis and falls. The short-term BP regulation warrants further investigation as BP drops during the transition from supine to an upright position may be implicated in the aetiology of falls in HD.

## Supporting information

S1 File Research dataset. (XLSX)

S1 Protocol Head-up tilt to 60 degrees (HUT-60°) protocol. (DOC)

S1 Table Heart rate variability (HRV) data of study participants. (DOCX)
