# Supplementary material for: Baroreflex function, haemodynamic responses to an orthostatic challenge, and falls in haemodialysis patients
Source: PLoS One. 2018 Dec 6;13(12):e0208127. doi: 10.1371/journal.pone.0208127 (PMC6283578; doi:10.1371/journal.pone.0208127)
Supplement: S1 Protocol — (DOC) [file pone.0208127.s002.doc]

**Head-Up Tilt to 60 degrees (HUT-60^o^) protocol**

1. The assessment room will be private, quiet, of adequate size for all equipment, well ventilated with a constant temperature of approximately 20-22°C and humidity < 60%.
2. The assessment room is located within the Monklands Hospital renal dialysis unit and has immediate access to resuscitation equipment and medical staff trained in immediate life support.
3. Participants will be asked to have a light meal about 2-3 hours before testing, and they will be asked not to smoke or to consume caffeine/alcohol for at least 2 hours before the appointment. They will also be asked to avoid unaccustomed physical exercise or vigorous exercise in the 24 hours preceding testing.
4. Participants will attend on a non-dialysis day, ideally one day post dialysis, on their usual medication only.
5. Upon arrival, patients will be connected to the TFM as per manual instructions and helped onto the tilt table and will be secured in place by straps around their thighs and feet. There is a foot platform where they can rest their feet whilst in the HUT position.
6. The TFM continuous blood pressure monitor will be kept at heart level throughout the measurement (as per manual instructions) in order to correct the hydrostatic effects of head-up tilting. The monitor will be fastened to the patient’s forearm by means of a Velcro fastener, and the patient’s arm will be supported by an arm sling that will secure the monitor at heart level during the HUT-60°.
7. Patients will be instructed not to talk or sleep during the procedure, and that they can request to stop at any point if they wish. Patients will also be asked to remain as still as possible (avoid isometric muscular contractions) during the whole duration of the procedure
8. Following patient set up, patients rest horizontally for 10 minutes before baseline measurements are taken.
9. Baseline measurements in the supine position are taken for a further 5 min.
10. The Tilt table is then inclined with a smooth transition and all haemodynamic data is recorded (from the start of transition) for a period of 5 minutes on 60 degrees head up (60 degrees is the recommended tilt angle for activation of baroreflex), if no symptoms develop.
11. If symptoms develop during this 5 minute period (see Termination Criteria), the patient is immediately returned to the supine position and nature of symptoms recorded from a list.
12. Patients will then return to horizontal supine position and remain there for 5 minutes. During this period, all physiological data will continue to be recorded.
13. Patients will be supervised in the assessment area until all indices of cardiovascular function have stabilised to pre-testing levels.

**Summary of patient time with TFM**

|  | **Time period (min)** | **Action** |
| --- | --- | --- |
| 1. | 10 | Preparation/set up |
| 2. | 10 | Rest period in the supine position |
| 3. | 5 | Baseline measurements taken in the supine position |
| 4. | 5 | Measurements taken whilst at 60 degrees HUT |
| 5. | 5 | Return to supine position |
| 6. |  | Supervised until all physiological measures return to baseline figures |
|  |  |  |

**Exclusion criteria for HUT**

- Unable/refuses to provide informed consent
- ˂18 years or>90 years
- Non ambulatory or lower limb amputee without prosthesis
- Unstable on dialysis and medications treatment (e.g. volume overloaded or uncontrolled cardiac arrhythmias such as atrial fibrillation, severe AV blocks, SVTs)
- Unstable medical conditions (e.g. recent MI, or TIA, uncontrolled diabetes)
- Suspected or known aneurysm
- A known severe stenosis anywhere (e.g. heart valves, LV outflow obstruction, coronary or carotid or cerebrovascular artery stenosis)
- pregnancy

**Test Termination Criteria**

The test will be terminated and the patient will be laid flat immediately and alarm will be raised if any of the following occurs:

1. Induction of syncope or presyncope symptoms associated with marked hypotension or bradycardia or both (dizziness, palpitations, blurred vision, nausea, lightheadedness, headache)
2. Completion of planned duration of test
3. Patient distress or discomfort
4. Patient request
5. Acute malignant arrhythmia (AF, SVT, HR<40bpm)
6. SBP falls >80mmHg or falls rapidly
7. Substantial reductions in BP (SBP>20 mmHg and/or DBP>10mmHg) compared to their baseline values without compensatory changes in other heamodynamic variables (HR, peripheral vascular resistance, stroke volume, cardiac output). This is defined as orthostatic syncope, where there are no compensatory adjustments due to failure of sympathetic tone to increase.
8. HR rises to greater than 170/min
9. Chest pain
10. Asystole
11. Any other adverse/unexpected event develops

The test will be interrupted immediately if any of the above criteria develop and the participant will be returned to the supine position. Loss of consciousness will be prevented by continuous monitoring of HR and BP and termination of the test when both start decreasing substantially, without any other observations of compensatory mechanisms. A tilt duration of 20- 45 minutes has become widely accepted within the literature. However, these studies aim to induce syncope and use results diagnostically. The aim of this study is to gain an index marker of BRS and so a timeframe of 5 minutes tilt will be used.
