# Supplementary material for: Baroreflex function, haemodynamic responses to an orthostatic challenge, and falls in haemodialysis patients
Source: PLoS One. 2018 Dec 6;13(12):e0208127. doi: 10.1371/journal.pone.0208127 (PMC6283578; doi:10.1371/journal.pone.0208127)
Supplement: S1 Table — (DOCX) [file pone.0208127.s003.docx]

**Supporting information file 3.** Heart rate variability (HRV) and blood pressure variability (BPV) characteristics of study participants (mean ± standard deviation).

| **Variables** | **All patients** | **Fallers** | **Non-fallers** | **P-value** |
| --- | --- | --- | --- | --- |
| **Heart rate variability** |  |  |  |  |
| LFnu-RRI (%) | 42.1±21.8 | 44.8±21.1 | 38.5±22.8 | 0.192 |
| HFnu-RRI (%) | 57.9±21.8 | 55.2±21.1 | 61.5±22.7 | 0.192 |
| VLF-RRI (ms^2^) | 123.8±250.2 | 120.1±273.7 | 128.9±217.9 | 0.136 |
| LF-RRI (ms^2^) | 119.7±342.8 | 135.5±437.7 | 97.5±125.8 | 0.143 |
| HF-RRI (ms^2^) | 234.8±753.8 | 178.8±584.6 | 313.8±949.4 | 0.007 |
| PSD-RRI (ms^2^) | 478.3±1175.6 | 434.4±1251.6 | 540.2±1079.6 | 0.040 |
| LF/HF-RRI (1) | 1.5±2.7 | 1.4±1.5 | 1.7±3.8 | 0.192 |
| LF/HF (1) | 0.9±1.9 | 08±0.5 | 1.2±2.9 | 0.271 |
| **Blood pressure variability - systolic** |  |  |  |  |
| LFnu-sBP (%) | 29±13.1 | 26.8±11.3 | 31.7±15.1 | 0.126 |
| HFnu-sBP (%) | 29.3±17.7 | 32±19.7 | 24.9±13.6 | 0.132 |
| VLF-sBP (mmHg^2^) | 6.2±16.3 | 4.8±7.3 | 8.3±23.8 | 0.424 |
| LF-sBP (mmHg^2^) | 4.8±14.3 | 3±4.3 | 7.3±21.5 | 0.461 |
| HF-sBP (mmHg^2^) | 3.6±10.6 | 2.7±2.8 | 4.9±16.1 | 0.790 |
| PSD-sBP (mmHg^2^) | 14.5±40.6 | 10.4±12.6 | 20.5±61 | 0.515 |
| LF/HF-sBP (1) | 1.6±2.6 | 1.2±0.9 | 2.1±3.9 | 0.163 |
| LF/HF (1) | 0.9±2 | 0.7±0.6 | 1.2±3 | 0.995 |
| **Blood pressure variability - diastolic** |  |  |  |  |
| LFnu-dBP (%) | 30.4±11.2 | 29.8±10.1 | 30.9±12.7 | 0.701 |
| HFnu-dBP (%) | 28.9±16.3 | 28.3±15.7 | 28.9±17.2 | 0.866 |
| VLF-dBP (mmHg^2^) | 2.3±3.9 | 2.2±4 | 2.4±3.8 | 0.249 |
| LF-dBP (mmHg^2^) | 1.5±1.8 | 1.4±1.9 | 1.7±1.7 | 0.126 |
| HF-dBP (mmHg^2^) | 1.4±2.5 | 1.1±1.5 | 1.8±3.4 | 0.123 |
| PSD-dBP (mmHg^2^) | 5.2±6.7 | 4.7±6.7 | 5.9±7 | 0.088 |
| LF/HF-dBP (1) | 1.6±1.5 | 1.5±1.2 | 1.6±1.9 | 0.926 |
| LF/HF (1) | 0.9±1.8 | 0.7±0.5 | 1.1±2.7 | 0.413 |

**Abbreviations**: LFnu-RRI: Normalized low frequency component of HRV; HFnu-RRI: Normalized high frequency component of HRV; VLF-RRI: Very low frequency component of HRV; LF-RRI: Low frequency component of HRV; HF-RRI: High frequency component of HRV; PSD-RRI: Power spectral density of HRV; LF/HF-RRI: Low frequency/high frequency ratio of HRV; LF/HF: Low frequency-diastolic blood pressure/HF-RRI; LFnu-sBP: Normalized low frequency component of systolic BPV; HF-nu-sBP: Normalized high frequency component of systolic BPV; VLF-sBP: Very low frequency component of systolic BPV; LF-sBP: Low frequency component of systolic BPV; HF-sBP: High frequency component of systolic BPV; PSD-sBP: Power spectral density of systolic BPV; LF/HF-sBP: Low frequency/high frequency ratio of systolic BPV; LFnu-dBP: Normalized low frequency component of diastolic BPV; HF-nu-dBP: Normalized high frequency component of diastolic BPV; VLF-dBP: Very low frequency component of diastolic BPV; LF-dBP: Low frequency component of diastolic BPV; HF-dBP: High frequency component of diastolic BPV; PSD-dBP: Power spectral density of diastolic BPV; LF/HF-dBP: Low frequency/high frequency ratio of diastolic BPV.
